# In vivo co-registered hybrid-contrast imaging by successive photoacoustic tomography and magnetic resonance imaging

**DOI:** 10.1016/j.pacs.2023.100506

**Published:** 2023-05-08

**Authors:** Shuangyang Zhang, Zhichao Liang, Kaiyi Tang, Xipan Li, Xiaoming Zhang, Zongxin Mo, Jian Wu, Shixian Huang, Jiaming Liu, Zhijian Zhuang, Li Qi, Wufan Chen

**Affiliations:** a School of Biomedical Engineering, Southern Medical University, Guangzhou, Guangdong, China; b Guangdong Provincial Key Laboratory of Medical Image Processing, Southern Medical University, Guangzhou, Guangdong, China; c Guangdong Province Engineering Laboratory for Medical Imaging and Diagnostic Technology, Southern Medical University, Guangzhou, Guangdong, China

**Keywords:** Photoacoustic Tomography, Magnetic resonance imaging, Co-registration, Hardware-software solution, Multi-scale characteristics

## Abstract

Magnetic resonance imaging (MRI) and photoacoustic tomography (PAT) offer two distinct image contrasts. To integrate these two modalities, we present a comprehensive hardware-software solution for the successive acquisition and co-registration of PAT and MRI images in in vivo animal studies. Based on commercial PAT and MRI scanners, our solution includes a 3D-printed dual-modality imaging bed, a 3-D spatial image co-registration algorithm with dual-modality markers, and a robust modality switching protocol for in vivo imaging studies. Using the proposed solution, we successfully demonstrated co-registered hybrid-contrast PAT-MRI imaging that simultaneously displays multi-scale anatomical, functional and molecular characteristics on healthy and cancerous living mice. Week-long longitudinal dual-modality imaging of tumor development reveals information on size, border, vascular pattern, blood oxygenation, and molecular probe metabolism of the tumor micro-environment at the same time. The proposed methodology holds promise for a wide range of pre-clinical research applications that benefit from the PAT-MRI dual-modality image contrast.

## 1 Introduction

Tomographic imaging of living animals has been an important task for preclinical research because it provides cross-sectional images of the subject without surgical intervention. This unique capability has differed it from other transmissive or reflectance imaging approaches such as whole-body fluorescence imaging [1] or digital radiography [2]. Among many tomographic imaging techniques, Photoacoustic Tomography (PAT) and Magnetic Resonance Imaging (MRI) are two advanced biomedical imaging modalities that have been used in various pre-clinical imaging applications ranging from tumor screening [3], [4], [5], [6], [7], therapy evaluation [8], [9], [10], to functional brain imaging [11], [12], [13], [14], [15], [16] and so on. In PAT, an image that maps the original energy deposition inside the target is formed by detecting and processing the ultrasonic signals generated by laser illumination [17], [18]. PAT is able to reveal the distribution of endogenous tissue absorbers, such as oxyhemoglobin (HbO_2_) and deoxyhemoglobin (Hb), and exogenous optical probes, such as the FDA-approved Indocyanine Green (ICG), by identifying their absorption spectrum under multiple wavelength excitations [19], [20], [21]. On the other hand, as a Nobel-winning technology, MRI provides cross-sectional images of the subject by using non-ionizing electromagnetic radiation and measuring the nuclear magnetic resonance signal, and thus enables multitudinous tissue contrast. MRI is able to provide comprehensive, multi-parametric information on anatomy, function and metabolism. Thanks to the emergence of diffusion MRI, functional MRI and other technologies, MRI has covered various clinical neurological, psychiatric, cardiac and abdominal applications [22].

Given their outstanding imaging capabilities, these two imaging modalities are complementary at multiple dimensions. Firstly, they have distinct image contrast mechanisms. PAT provides molecular imaging capability that reflects the optical characteristics of light absorbers inside tissue [23], [24], [25], whereas MRI images the density of hydrogen protons such that soft-tissue contrast is revealed. Secondly, the imaging speeds are complementary. Thanks to recent advancement in laser technology, PAT imaging speed could reach over 7000 frames per second [26], whereas high-field MRI system could only acquire at most one to two images per second without scarifying image quality [27]. Thirdly, their spatial resolutions are matched. Common spatial resolutions for commercial pre-clinical MRI and PAT scanners are both around tens to hundreds micrometres [28] given an imaging field of view of several square centimetres. Finally, PAT and MRI also share the advantages of being non-invasive, non-ionizing and label-free.

PAT-MRI dual-modality imaging that simultaneously acquires structural, functional, and molecular images has great potential to push the image analyse focus to multiple scales, allowing for much broader preclinical research impacts. Previous attempts to integrate images from these two modalities were confined to either rigid registration (e.g. imaging of rigid structures such as the brain [29], [30], [31]) or no registration [32], [33], [34]. Co-registration of abdomen PAT and MRI images of small animals using a customized single-use silicone MRI holder has been reported previously [35], which realized the registration of soft tissue images for the first time. Most recently, a proof-of-concept concurrent PAT-MRI imaging system has been proposed with phantom-based feasibility validation [36]. Development of such system requires high cost for customized instrumentation, and sacrifices the flexibility of individual system. Apart from hardware registration, robust software registration algorithms are required to precisely align the images from different modalities. Co-registration of PAT and MRI images of the brain of small animals has been proposed [29]. It combines mutual information based rigid registration algorithm with manually labelled anatomical landmark for the matching of the brain, which is a non-deformed object.

Although there were these aforementioned early demonstrations of successive or concurrent PAT-MRI imaging for various applications, implementing a rigidly co-registered, dual-modality imaging solution faced significant challenges. First, PAT imaging requires coupling media (e.g. water) whereas MRI imaging does not. The purpose of the coupling media is to let the excited ultrasound to propagate. However, during modality switching, this coupling media will inevitably affect the posture of the animal. Second, MRI requires a strictly no-metal imaging environment, making the design and fabrication of a robust bimodal registration tool difficult. Third, it requires spatial resolution matching at the axial, radial, and tangential directions, simultaneously. Fourth, flexible, easy-to-use software compensation algorithms for dual-modality image registration are required for high repeatability imaging experiments. Fifth, similar to the attenuation correction in a Positron Emission Tomography/Computed Tomography (PET/CT) scanner where CT image is used to enhance PET imaging, the structural information in MRI might be used to correct for image degradation in PAT in order to excavate deeper information [37]. Finally, the obtained dual-modality images from long-term in vivo longitudinal imaging should be validated, and the performance of the whole imaging protocol should be benchmarked and analysed.

Here, we propose a method for the successive acquisition and co-registration of PAT and MRI data in in vivo mice studies. The method includes a novel dual-modality imaging bed and a robust dual-modality spatial co-registration protocol. The 3D-printed imaging bed is specifically designed to secure the posture and position of the animals during modality switching. Based on this bimodal imaging bed, we design a rigorous data acquisition procedure, a stable modality switching protocol, and a highly automated data processing software suite to enable precise matching of the dual-modality images of the entire animal body. We demonstrate the excellent capability of this successive PAT-MRI dual-modality imaging method in in vivo applications including tomographic hybrid contrast observation of important organs (simultaneously structural, functional, and molecular imaging) and multi-timescale longitudinal monitoring of tumor development (from minute-scale drug uptake to week-long evolution of tumor size and hypoxia condition). This integrated and standardized protocol for in vivo small animal PAT-MRI dual-modality imaging will help unlock and promote even more preclinical research applications of these two modalities, such as simultaneous functional-anatomical brain imaging, bimodal contrast agent development, and anatomically specific pharmacokinetic research and etc.

## 2 Method

We first describe the proposed co-registered successive PAT-MRI imaging solution. Our solution is developed particularly for a 7-tesla MRI scanner (Pharmascan, Burker, Germany) and a commercial multispectral cross-sectional PAT system (MSOT inVision128, iThera Medical, Germany), both of which are among the most popular commercial imaging platforms for pre-clinical small animal imaging. The radial resolution of the MRI and PAT systems were similar (∼ 150 µm). The axial resolution of MRI could reach < 100 µm, but for the PAT it is limited to 800 µm. However, the axial resolution of MRI is tunable such that the spatial resolutions, both axial and radial, are matched for the two modalities. To ensure precise spatial co-registration, our solution includes a specially designed dual-modality imaging bed, a rigorous data acquisition procedure, and a highly automated data processing software.

### 2.1 Design of the PAT-MRI dual-modality imaging bed

**Fig. 1:**
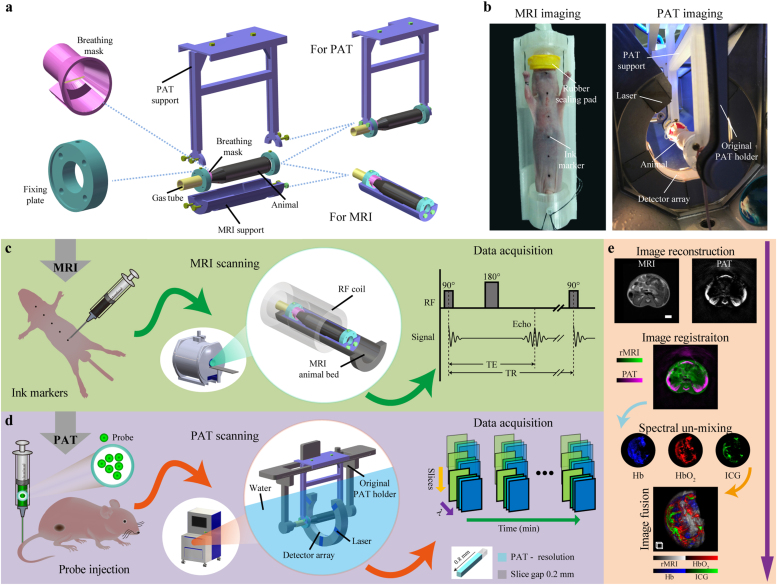
Co-registered hybrid-contrast imaging of living mice by successive PAT and MRI. (a) The dual-modality imaging bed includes a PAT support, an MRI support, a gas tube, a breathing mask and two fixing plates. Image acquisition for the two modalities can be realized by assembling different components. (b) Photographs of a nude mouse placed on the animal bed before MRI and during PAT imaging. (c) MRI image acquisition process. Axial marker assignment: the skin surface of nude mice is marked with Chinese ink. MRI scanning: after assembling the MRI support, the animal was placed on the animal bed and transferred to the centre of the magnet for scanning. MRI data acquisition: a 2-D spin echo sequence was used to acquire MRI images. (d) PAT image acquisition process. Probe injection: optional exogenous probe such as ICG was injected through the tail vein. PAT scanning: after assembling the PAT support, the animal was loaded into the PAT imaging chamber filled with water for scanning. PAT data acquisition: 5-D multi-wavelength multi-slice longitudinal PAT images are acquired. (e) Image processing process. This process includes image reconstruction, image co-registration, spectral un-mixing, and image fusion. Scale bars, 3 mm. Scale box, 1 mm^3^. Details of the dual-modality imaging bed are shown in Video 1.

The major purpose of the dual-modality animal imaging bed is to ensure that the animal maintains at the same positioning and posture during successive PAT and MRI imaging. Because the animal has to be bathed in water during PAT imaging, the biggest challenge is to make sure the animal position does not change during the switching between the two imaging modalities. This is achieved by designing the animal bed according to the PAT and MRI system environment and geometric dimensions. The dual-modality imaging bed can be separated into two parts, one for PAT, and the other for MRI [Fig. 1(a)]. All the components except the gas tube are 3D-printed with polylactic acid (PLA) material using a desktop 3D printer (JennyPrinter 4th Generation, Jenny Technology Co., Ltd, China), the components include:

#### 2.1.1 Gas tube

A 10 mm diameter tube that connects the anaesthetic port to the breathing mask. The gas tube is made of rubber and therefore it can supply the anaesthetic gas to the animal while preventing water from entering the mask.

#### 2.1.2 Breathing mask

The breathing mask functions like a swimming goggle except that it only covers the mouth and nose of the animal. It includes a funnel-shaped structure, a built-in copper wire, and a sealing silicone pad. One small end of the mask is connected to the anaesthetic port through the gas tube. The other end of the mask is sealed with a silicone pad with a small hole in the center, such that the frontal part of the mouse head fits into the hole. The copper wire is mounted transversely inside the breathing mask for hooking the teeth of the mouse during PAT imaging. In this way, the mouse face can be closely fitted to the silicone sealing pad, and drowning of the animal can be prevented. The mask also helps to keep the mouse head steady during imaging.

#### 2.1.3 Fixing plates

Both the left and right sides of the imaging bed contain the fixing plate. The functions of the two fixing plates include: 1) fixing the animal onto the PAT and MRI supports, and 2) binding the limbs of the mouse and fixing the breathing mask. These fixing plates can be firmly attached to the imaging supports by using plastic or copper screws, and then the arms and legs of the animal can also be tied to the support.

#### 2.1.4 PAT support

It includes two components: the mounting plate and the cantilever, which are assembled by screw combination. The mounting plate can be perfectly attached on the original animal holder of the PAT system, and can be translated in the axial direction of the nude mouse (less than 1 cm) to facilitate the connection of the gas tube. The cantilever is fastened to the fixing plates by screws such that the animal to be imaged is in a suspended state. This design centers the animal in the ring-shape detector, and lets none obstruction appear along the sound propagation path. In this way, the PAT image quality can be ensured.

#### 2.1.5 MRI support

A curved base-plate used to fix the animal during MRI imaging by screwing to the fixing plates. The MRI support is adapted to the body contour of the animal, prevents the animal from deformation, and matches the shape of the original MRI bed.

Supplementary material related to this article can be found online at doi:10.1016/j.pacs.2023.100506.

The following is the Supplementary material related to this article Video 1..

Video 1

### 2.2 Successive PAT-MRI dual-modality image acquisition process

The image acquisition process is divided into four steps.

#### 2.2.1 Axial marker assignment

Chinese ink is used as markers for axial registration because it can be visualized in both MRI and PAT. The ink is marked on the skin surface of nude mice using a Gauge 20 needle [Fig. 1(c)]. The marker size is less than 1 mm and the separation distance is 1 cm such that minimum interference is caused to the images.

#### 2.2.2 MRI imaging

The animal is fastened on the MRI support and transferred to the MRI imaging cavity, and the location of the mouse to be scanned is positioned in the center of the RF coil [Fig. 1(c)]. During MRI imaging, we use a 2-D spin-echo sequence to scan the axial image in the XY plane. After the acquisition is completed, the dataset is resliced along the XZ and YZ directions to obtain the coronal and sagittal images.

#### 2.2.3 Modality switching

First, we use screws to fasten the MRI support on the fixing plates. Then, we unscrew the screws on the cantilever and remove the PAT support. During this process, the body of the mouse is always in a tight state that ensure an unchanged posture. After the animal support is switched, exogenous contrast agents for PAT imaging such as ICG can be injected. A video showing the above processes is available in Video 1.

#### 2.2.4 PAT imaging

We fix the PAT support on the original animal holder of the PAT system, and connect the gas tube to the anaesthesia port on the holder [Fig. 1(d)]. The whole assembly is then transferred to the imaging chamber filled with distilled water pre-heated to 34-degree Celsius. The assembly is set on a built-in motorized translation stage, such that the animal can be positioned to the optimal field of view. Finally, multispectral 3-D PAT image acquisition is carried out. When the PAT imaging section is finished, the PAT support is taken out from the imaging chamber and the animal is released.

### 2.3 The image processing algorithm for PAT-MRI dual-modality imaging

**Fig. 2:**
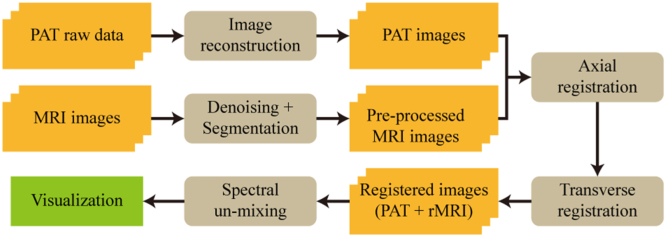
The image processing workflow for PAT-MRI dual-modality imaging. The brown boxes indicate the image processing operations. The yellow boxes indicate image data.

After the dual-modality imaging, processing of the acquired images is employed to refine the co-registration [Fig. 2].

#### 2.3.1 MRI image pre-processing

Each slice of the MRI data is pre-processed to remove noise, segment the subject from the background and resize the image. For denoising, we adopt a 2-D adaptive low-pass Wiener filter to eliminate random noise in the MRI images. For segmentation, we use the 3D slicer software [38] to manually segment the subject from the background. For image resizing, interpolation of the image background is performed around the image to change the image size to 30 × 30 mm^2^ to match that of the PAT images.

#### 2.3.2 PAT image pre-processing

In the iterative reconstruction of the PAT images, the pixel values of the PAT images are limited to be non-negative. The obtained non-negative PAT images suppress the background signal and highlighted the body contour of the animal. Based on these contrast-enhanced PAT images, we again use the 3D slicer software to segment the animal from the background in the PAT images.

#### 2.3.3 Axial registration with external markers

Before imaging, the surface of the mouse is marked with small markers using black Chinese ink because the ink markers can be visualized on both PAT and MRI. Because the axial resolution of the PAT is much lower than the MRI, each ink marker will appear on multiple PAT images. To determine the accurate axial position of the marker, we first quantify the mean intensity of the marker on all the PAT images with marker visible, and then apply Gaussian fitting to the obtained intensity curve. The peak position of the fitted curve is chosen as the true marker position. The PAT image nearest to this position is determined to be the image that corresponded to the marked MRI image (the marker is usually visible on only one MRI image). Then according to this image, the rest of the PAT images are aligned with the MRI images and axial registration is done. Following the same principle, multiple markers on the tissue surface can be used to further enhance axial registration accuracy.

#### 2.3.4 Transverse registration

After the images are axially registered, we perform transverse registration on corresponding PAT-MRI image slices by using an automated rigid registration algorithm based on mutual information (MI) [39], a similarity metric commonly used in multimodal image registration problems. The value of MI is larger when the two images share a high degree of similarity or overlap. It was calculated by the following equation:

(1) $$\mathrm{MI}\left(A,B\right)=H\left(A\right)+H\left(B\right)-H(A,B)$$

where $H\left(A\right)$ and $H\left(B\right)$ denote the entropy of images A and B (MRI and PAT images, respectively) and H(A,B) denotes the joint entropy of these images. This will compensate for the small shifting or rotation of the animal during modality switching, and further align the dual-modality images. The registration procedure is implemented using the built-in function ‘imregister’ in MATLAB (Mathworks). During registration, the PAT image is set as the reference image and the MRI image as the moving image. A rigid transformation (including translation and rotation) between the two images is estimated using the algorithm and applied to the MRI image for registration. In our implementation of the algorithm, the growth factor, minimum size and initial value of the search radius were set to be 1.05, 1.5 × 10^−6^ and 6.3 × 10^−3^, respectively, and the maximum number of optimizer iteration was 100.

#### 2.3.5 Spectral Un-mixing

The reconstructed PAT image$\;p\left(\lambda \right)$ is a combination of various endogenous and exogenous absorbers. There is a linear relationship between the PAT image $p\left(\lambda \right)$ and the absorption coefficient value $\mu_{a}^{i}\left(\lambda \right)$ of the identified chromophore *i*:

(2) $$p\left(\lambda \right)=\sum_{i}\mu_{a}^{i}\left(\lambda \right)=\sum_{i}c_{i}\varepsilon_{i}\left(\lambda \right)$$

where, $c_{i}$ is the concentration of chromophore *i* and $\varepsilon_{i}\left(\lambda \right)$ is the molar extinction coefficient at *λ* wavelength. For PAT images acquired with multispectral excitation, we perform spectral un-mixing to identify the distribution of endogenous (HbO_2_, Hb) or exogenous absorbers (such as ICG) from the background [Fig. 1(e)]. The un-mixing is based on a linear algorithm [21] with multispectral PAT images as inputs. After obtaining the concentrations of HbO_2_ ($c_{{\mathrm{HbO}}_{2}}$) and Hb ($c_{\mathrm{Hb}}$), oxygen saturation (SO_2_) can be computed as:

(3) $${\mathrm{sO}}_{2}=\frac{c_{{\mathrm{HbO}}_{2}}}{c_{{\mathrm{HbO}}_{2}+}c_{\mathrm{Hb}}}$$

### 2.4 Experimental setups

#### 2.4.1 Magnetic resonance imaging

All MRI scan were performed on a 7 T small animal MRI system (Pharmascan, Bruker, Germany) operated by ParaVision 6.0 software platform. A 1 H transmit-receive volume coil with 40 mm inner diameter was used for signal transmitting and receiving. The animal was anesthetized with 4% isoflurane (RWD, China). We assembled the MRI support, fixed the limbs of the anesthetized animal on the fixing plates on both sides, and hooked the teeth of the nude mouse on the copper wire in the breathing mask. In order to reduce the image artefacts caused by respiratory movements, medical oxygen mixed with 1% isoflurane was transmitted through the gas tube to the breathing mask, so that the respiratory rate of nude mice was maintained at 15–20 times/min. The body temperature of the nude mouse was monitored using the rectal probe of a small animal monitoring system (Model 1030, Small Animal Instruments Inc., New York, NY, USA), and stabilized at 37 ± 0.1 °C using the heater module.

The T2 MRI images of nude mouse were obtained using a 2-D spin echo sequence (Turbo rapid acquisition with refocused echoes) with the following imaging parameters: RARE factor 8, echo time 10 ms, repetition time 6000 ms, 5 averages, slice thickness/gap 0.8/0.2 mm, field of view 25 × 25 mm^2^, matrix 250 × 250, spatial resolution 0.1 × 0.1 × 0.8 mm^3^. Sagittal T2 images of the YZ plane where the markers located were firstly acquired. Based on these images, the slice direction and position of the axial image was selected so that each marker was at the centre of the slice and the slice direction was perpendicular to the long-axis of the animal. The axial T2 images covering either the upper or lower parts of nude mouse were then acquired because the coil's effective imaging range was insufficient to cover the entire mouse.

#### 2.4.2 Photoacoustic tomography imaging

A commercial small animal multispectral photoacoustic tomography system [MSOT inVision128, iThera Medical, Germany, Fig. 1(d)] was employed to perform all the PAT imaging. A pulsed OPO laser (670–960 nm tunable) with pulse width < 10 ns, repetition rate of 10 Hz and a peak pulse energy of 60 mJ at 760 nm is employed in this PAT system. The laser light provides a diffused, homogeneous, and 360-degree illumination over the surface of the animal. The generated ultrasonic waves are detected by 128 toroidally focused ultrasound transducers with a centre frequency of 5 MHz (60% bandwidth) arranged over an azimuth span of 270-degrees around the cylinder with a radius of curvature of 41 mm [Fig. 1(d)].

When switching to PAT imaging, we needed to assemble the PAT support on the fixing plates with screws first, and then removed the MRI support. This is to ensure that the posture of the animal did not changed during the modality switching process. Finally, the animal-fixed PAT support was attached to the original PAT holder which aligned it with the center of the transducer and immersed the animal in 34 °C heated water for ultrasonic coupling and warm keeping.

All nude mice were anesthetized with 1% isoflurane during imaging. Whole body imaging was realized by translating the PAT support axially with the built-in motorized translation stage. For contrast-enhanced PAT, an insulin injection needle was embedded into the tail vein in advance, and was connected to a long Polyethylene Tubing 10 (PE 10) that enabled contrast agents injection (such as ICG) from outside the imaging chamber. Multispectral PAT images were acquired at five different illumination wavelengths: 700, 730, 760, 800 and 850 nm and averaged with signals from 10 frames per wavelength. The ultrasound time series signals are then reconstructed into 2D pressure maps using a model-based iterative reconstruction algorithm with a 30 × 30 mm field of view at 300 × 300 pixels [18]. Finally, the linear un-mixing algorithm was used to calculate the distribution of HbO_2_, Hb and ICG.

#### 2.4.3 Animal models

All animal experiments were approved by the local Animal Ethics Committee of Southern Medical University and were performed in accordance with current guidelines. In the in vivo animal imaging experiment, 6 healthy nude mice (12–15 g/each, female, Southern Medical University, Guangzhou, China), and 4 nude mice carrying 4T1 mammary carcinoma (Southern Medical University Cancer Institute, Guangzhou, China) were used. Animals were kept in ventilated cages inside a temperature-controlled room, under a 12-h dark/light cycle. In order to reduce abdominal peristalsis artifacts caused by food digestion and to prevent the mice from excreting and polluting the imaging environment during PAT imaging, the nude mice were fasted for 8 h before imaging.

#### 2.4.4 Evaluation of the registration accuracy

The co-registration accuracy of the dual-modality images was analyzed by calculating the Dice Similarity Coefficient (DSC), which was defined as:

(4) $$\mathrm{DSC}=\frac{2\left|X\cap Y\right|}{\left|X\right|+\left|Y\right|}$$

DSC made quantification of the overlap between two binary masks *X* and *Y* (initial PAT subject mask and registered MRI (rMRI) subject mask obtained from the segmentation). A higher DSC value means a better overlap between the two binary masks, i.e., a more accurate image registration performance.

Moreover, we calculated the structural similarity (SSIM), which measured the similarity between the PAT image and the rMRI image in terms of brightness (mean image intensity), contrast (intensity variance) and structure (intensity covariance). It is defined as:

(5) $$\mathrm{SSIM}=\frac{\left(2\mu_{I}\mu_{x}+C_{1}\right)\left(2\sigma_{I\cdot x}+C_{2}\right)}{\left(\mu_{I}^{2}+\mu_{x}^{2}+C_{1}\right)\left(\sigma_{I}^{2}+\sigma_{x}^{2}+C_{2}\right)}$$

where, $\mu_{I}$ and $\mu_{x}$ are the mean of the rMRI image *I* and the restored image *x*, respectively. $\sigma_{I}^{2}$ and $\sigma_{x}^{2}$ are the variance of *I* and *x*, respectively. $\sigma_{I∙x}$ is the covariance of the image *I* and *x*. The small constants $C_{1}$ and $C_{2}$ are given by:

(6) $$C_{1}={\left(K_{1}L\right)}^{2},C_{2}={\left(K_{2}L\right)}^{2}$$

where, $K_{1}$ and $K_{2}$ are set as $K_{1}=0.01$ and $K_{2}=0.03$ respectively.

## 3 Results

To demonstrate the feasibility of our proposed solution in in vivo applications, we implemented three-dimensional co-registered anatomical imaging, week-long longitudinal tumor monitoring, and temporal anatomical-molecular imaging, respectively.

### 3.1 Co-registered anatomical imaging by PAT and MRI

**Fig. 3:**
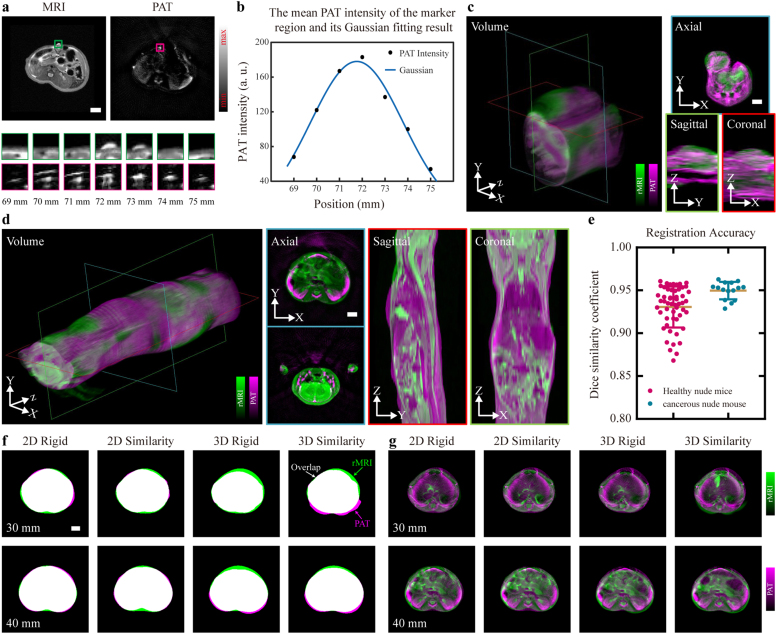
Spatial co-registration results. (a) The MRI and PAT images at 72 mm and the enlarged marker images at different axial positions. (b) The mean PAT intensity of the marker region at different axial position and its Gaussian fitting result. (c) The co-registered PAT-MRI dual-modality images from tumorous nude mouse. (d) The co-registered PAT-MRI dual-modality images from healthy nude mouse. All sagittal and coronal images are resampled and interpolated from the XY plane image stack, and display in 3D as volume images. (e) Quantification of the co-registration accuracy in DSC using image data from (c) and (d). (f) Overlays of body contours from the PAT-rMRI image pairs. (g) Overlays of the PAT-rMRI image pairs. All images in (c), (d), (f) and (g) are displayed with pseudo-color overlay (magenta for PAT and green for MRI). 2D: 2D co-registration. 3D: 3D co-registration. Rigid: rigid transformation consisting of translation and rotation. Similarity: nonreflective similarity transformation consisting of translation, rotation, and scale. Scale bars, 3 mm.

With the proposed successive PAT-MRI dual-modality imaging method, we achieved three-dimensional co-registered anatomical imaging of tumorous and healthy mice in vivo. Fig. 3(a) shows the corresponding PAT and MRI images at the ink marker position. In the MRI image, the ink marker can be localized easily, however, in the PAT image, the marker spreads for nearly 5 mm along the axial direction [Fig. 3(a)] due to its strong optical absorption, making the identification of the correct PAT slice difficult. To tackle this, we quantified the intensity of the marker at each slice, as plotted in Fig. 3(b), and then applied Gaussian fitting to the plot. The PAT image at the peak position of the fitted curve was considered to be the correct image that matched the MRI image. Figs. 3(c) and 3(d) show the co-registration results of the tumorous and healthy nude mouse respectively. By resampling and interpolating the XY plane image stack, we obtained the sagittal and coronal images and displayed them in 3D as volume images. The joint visualization of the PAT and MRI images reveals successful matching of the internal structure and the consistency of the body shapes of the animal. The kidneys, spleen and spine in the axial image of healthy nude mice are correctly overlapped, and the body contours are also consistent. Finally, we perform quantitative evaluation of the registration performance [Fig. 3(e)] using the DSC, which measures the percentage of the overlap between the two images. The average DSCs for healthy and tumorous mice are 93.06% and 95.12% respectively, demonstrating very high overlap between the PAT and MRI images.

To demonstrate the necessity of our axial registration method, we performed 3D co-registration of PAT-MRI dual-modality images without the help of the external markers for comparison. In addition, we also employed the similarity (consisting of translation, rotation, and scale) transformation besides the rigid transformation (consisting of translation and rotation) for image registration. The comparison results are shown in Fig. 3(f, g), and the quantitative analysis results of the co-registration accuracy are listed in Table 1. Compared with 3D co-registration, 2D co-registration results showed a better coincidence of body contours [Fig. 3(f)]. The DSCs for rigid and similarity of 3D co-registration results (0.928 ± 0.0264 and 0.9268 ± 0.0406) are lower than that of the 2D co-registration (0.9653 ± 0.0144 and 0.9625 ± 0.0134). Moreover, as shown in Figs. 3(g), 3(D) co-registration results showed dislocation of spine and surface veins at 30 mm and kidney misalignment at 40 mm, whereas in 2D co-registration results, these problems were solved or improved. Consistent with the previous results, the SSIM of 3D co-registration is lower than that of 2D co-registration, and the rigid transformation achieved the highest SSIM value (0.999 ± 0.0004). Since the 2D rigid transformation gives the best registration results, we have applied this registration method to all the experimental data.

**Table 1:** Quantitative analysis of the co-registration accuracy obtained by using different methods.

Metrics	2D co-registration		3D co-registration	
	Rigid	Similarity	Rigid	Similarity
DSC	**0.9653 ± 0.0144**	0.9625 ± 0.0134	0.928 ± 0.0264	0.9268 ± 0.0406
SSIM	**0.999 ± 0.0004**	0.9986 ± 0.0005	0.9985 ± 0.006	0.9985 ± 0.0006

### 3.2 Hybrid-contrast longitudinal recording of tumor growth

To demonstrate the capability of simultaneously anatomical and functional imaging, PAT-MRI dual-modality longitudinal observation on one nude mouse bearing 4T1 tumor was performed. The successive imaging was carried out on day 3, 5, 7, 9, 11, 13 and 17 post tumor implantation. MRI-T2 images that represent structural information and HbO_2_, Hb, HbT images that show hypoxia microenvironment obtained from spectrally un-mixed multispectral PAT images. The quantitative analysis results of the co-registration accuracy are listed in Table 2. To facilitate better visualization, we used the registered MRI images as structural priori to manually segment the tumor.

**Table 2:** Quantification of the co-registration accuracy during week-long longitudinal tumor monitoring.

Date	Day 3	Day 5	Day7	Day9	Day 11	Day 13	Day 17
DSC	0.9466 ± 0.0071	0.958 ± 0.0069	0.9298 ± 0.0049	0.939 ± 0.0147	0.9335 ± 0.0065	0.9456 ± 0.0039	0.9497 ± 0.0117

**Fig. 4:**
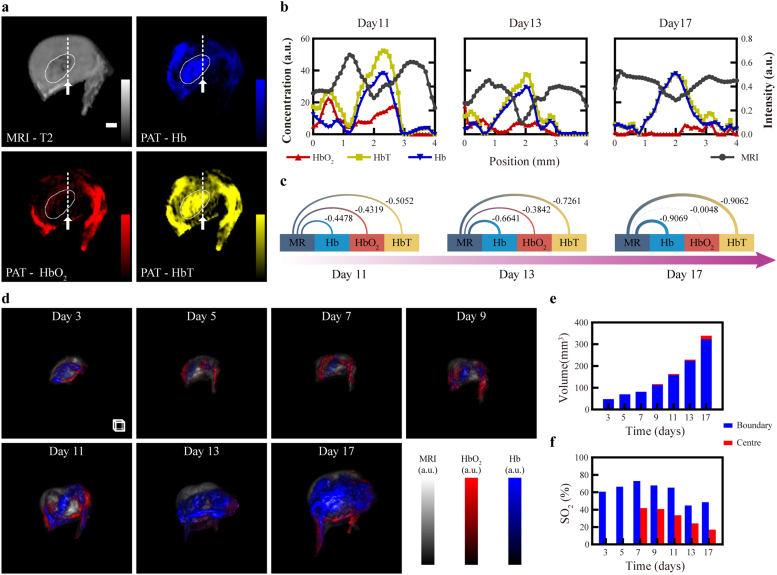
Week-long longitudinal monitoring of tumor dimension and hypoxia microenvironment in one living nude mouse by PAT-MRI imaging. (a) MRI image and PAT-derived Hb, HbO_2_ and HbT images in the central XY plane of the 4T1 tumor. The white solid-line area depicts the regions with similar or opposite features. (b) Image profiles drawn along the straight white dashed-lines in (a). (c) Pearson correlation coefficients of the profiles between the Hb, HbO_2_, HbT images and the MRI image. (d) 3D fusion display of MRI images and Hb, HbO_2_ images in longitudinal imaging of the 4T1 tumor. We delineated the mask of hypoxic regions inside the tumor and computed the volume size (e) from MRI images and the SO_2_ (f) from PAT images in the centre and boundary of the tumor separately. Scale bar, 1 mm. Scale box, 1 mm^3^. See also Video 2.

As shown in the dual-modality images taken at day 17 [Fig. 4(a)], the MRI-T2 image showed highly corresponding tumor geometry that matched the distribution of Hb, HbO_2_, and HbT. The white solid-line area depicts the regions with consistent or diametrically opposite features on these images (darker in the T2 and brighter in the Hb and HbT). T2 signal represents changes in blood oxygenation, and weakens when the concentration of Hb inside the tumor increases. To further analyze the correlation between the obtained structural and functional information, profiles [Fig. 4(b)] of the Hb, HbO_2_, and HbT images were obtained along the white dashed-lines [Fig. 4(a)] and then compared with that of the MRI image to calculate the Pearson Correlation Coefficient (PCC) [Fig. 4(c)]. The PCC values between T2 and Hb, HbO_2_ and HbT are − 0.9069, − 0.0048 and − 0.9062, respectively. The profiles show opposite spatial trends between T2 and Hb, HbT across the tumor, and the PCC values close to − 1 further illustrate the negative correlation of this spatial variation. Overall, the inspection of T2 and Hb revealed the existence of negative correlation, which became higher with time [Fig. 4(c)].

Moreover, as shown in a series of 3D fusion display using T2, Hb and HbO_2_ images obtained during the tumor development in Fig. 4(d), the growth of the tumor was accompanied by the development of neovasculature and the increase of tumor dimension. We also measured the change of tumor volume [Fig. 4(e)] from the MRI images and calculated the values of tumor SO_2_ [Fig. 4(f)] from the distribution of Hb and HbO_2_. Quantitative parameters obtained from the PAT images indicated that during tumor growth, the SO_2_ increased continuously from 60.67% on day 3–72.96% on day 7 for the whole tumor area and decreased from 40.68% on day 9–17.08% on day 17 for the tumor center alone. The overall volume of the tumor reached a 6-fold increase from 47.73 mm^3^ on day 3–339.75 mm^3^ on day 17. In addition, the volume of the central region of the tumor saw a 40-fold increase from 0.44 mm^3^ on day 7–17.7 mm^3^ on day 17. This experiment demonstrated the unique capability of label-free, long-term structural and functional hybrid contrast imaging of the PAT-MRI bimodal imaging method.

Supplementary material related to this article can be found online at doi:10.1016/j.pacs.2023.100506.

The following is the Supplementary material related to this article Video 2..

Video 2

### 3.3 Spatially localized high-speed imaging of molecular probes

**Fig. 5:**
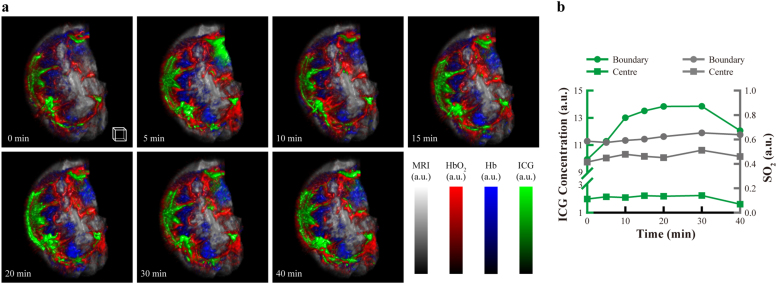
Temporal anatomical-molecular imaging of ICG perfusion inside 4T1 tumor. (a) 3D fusion display of the Hb, HbO_2_, ICG distributions on top of a co-registered MRI image of the 4T1 tumor microenvironment. PAT images were acquired at a 5-minute interval. (b) Time trace of the ICG concentration and SO_2_ of the hypoxic centre and boundary region of the tumor. Scale box, 1 mm^3^. See also Video 3.

To harness the high-speed imaging capability provided by PAT imaging, we applied intravenous (IV) injection of 200 μl of ICG (0.05 μg/μl) on a day-21 4T1 tumorous mouse after MRI imaging, and then performed PAT temporal imaging of the mouse at 5-minute intervals for 40 mins. ICG, which is an FDA-approved NIR fluorochrome commonly used as a contrast agent in retinal and tumor imaging, is able to metabolize in blood-rich organs and excreted into the bile within one hour [40], [41], [42]. Fig. 5(a) shows the volumetric images of MRI-T2, HbO_2_, Hb and ICG at various time points. As can be seen, almost immediately after the injection, a small amount of ICG signal appeared in the tumor. Then the signal gradually increased, indicating fast deposition of the ICG inside the tumor. Nevertheless, ICG only appeared in the boundary region of the tumor, and its concentration in the central region was relatively low across the whole imaging period. This indicated a hypoxic area had been developed in the center of the tumor. Furthermore, we measured the ICG concentration at different time at both the centre and boundary regions of the tumor, and the result was shown in Fig. 5(b). As can be seen, after ICG injection, there was no significant change in its concentration around tumor centre (< 5% fluctuation). However, the ICG concentration in the boundary kept on rising around the first 20 min and reached a peak increase of 39.12% at t = 20 min, and then slowly went down.

Supplementary material related to this article can be found online at doi:10.1016/j.pacs.2023.100506.

The following is the Supplementary material related to this article Video 3..

Video 3

## 4 Discussions

Successive PAT-MRI imaging saves the flexibility of individual modality, but the imaged animal is easily deformed during modality switching, making image registration problem more challenging. The presented work provides a unique strategy for meeting this challenge. To ensure the posture and position of the animal during modality switching, our successive PAT-MRI imaging method involves a hardware registration device and a software toolset with automatic processing capability.

On the hardware side, a novel small animal dual-modality imaging bed was designed. This 3D-printed imaging bed solves the water coupling problem in PAT imaging and ensures that the animal does not deform or displace while switching between MRI and PAT. Its introduction preserves the consistency in the shape of the entire mouse body or local lesion contour such as tumor boundary, and thereby simplifies the multi-modality image co-registration problem into a rigid registration problem solvable by standard image processing algorithms. Moreover, animal fastening method of the designed imaging bed was similar to that of the original PAT system. Therefore, PAT imaging artefacts induced by the bed were minimized and animal preparation time was not increased. The use of the breathing mask allowed for the animal to breathe freely underwater during PAT imaging and ensured a high survival rate of the animals. Also, assembling and disassembling procedures of the MRI/PAT supports were designed to be both convenient and stable, such that changes in animal pose and position were subtle. The animal bed is simple to manufacture, low cost, reusable, and compatible with the harsh MRI environment.

On the software side, an axial registration method based on external dual-modality markers and a transverse co-registration algorithm based on mutual information between MRI and PAT was developed to further improve image co-registration performance. The Chinese ink marked on the animal surface is not only minimum invasive and harmless, but also has PAT-MRI dual contrast. With the above unique advantages, our proposed dual-modality imaging strategy offers a unified, standardized, and convenient solution to implement successive acquisition of PAT and MRI images for in vivo preclinical animal research.

Furthermore, the feasibility of the proposed strategy was demonstrated in various dual-modality imaging scenarios. Firstly, healthy nude mouse and cancerous nude mouse spatial co-registration results showed high overlap of animal anatomy on the two images, illustrating the robustness and favourable performance of the proposed strategy. Secondly, dual-modality characterization of spatial and temporal heterogeneities of the hypoxia tumor microenvironment visualized vascular pattern changes throughout the entire tumor development period, revealing the possibility of label-free, multi-contrast monitoring of cancer development. Thirdly, high speed spatial and temporal tomographic imaging of exogenous contrast agent uptake inside tumor demonstrated highly accurate structural localization of the imaging probe, allowing for the study of drug metabolism dynamics with high spatial specificity. This preliminary demonstration of spatially localized continuous monitoring of contrast agent reveals the potential of our proposed method for structural enhanced dynamic molecular imaging.

Based on our work, we envision vast applications by the proposed successive PAT-MRI imaging technology. For example, some studies have shown that the central hypoxia of solid tumors is related to prognosis and treatment resistance [43], [44]. Therefore, longitudinal quantitative analysis of SO_2_ and HbT with PAT imaging, which is able to access the hypoxic micro-environmental changes, and longitudinal tumor morphology observation with MRI imaging, which can monitor tumor dimension and growth rate, can work together to provide a platform for the in vivo and *ex vivo* evaluation of anticancer therapies aimed at reducing hypoxia and inhibiting tumor angiogenesis [44], [45].

There are still some limitations in our study. Firstly, in routine MRI scans, the respiratory monitoring system is usually used to detect plateaus of animal respiration to overcome respiratory motion artifacts. However, for PAT-MRI dual-modality imaging, this respiratory monitoring gasket cannot be used because it has to be tightly fixed on the animal's abdomen and this will inevitably cause body deformation, making PAT-MRI co-registration difficult. Secondly, we acquired only MRI-T2 images to reveal animal anatomical information. Future work will focus on the integration of advanced MRI technologies such as functional MRI, quantitative susceptibility mapping, and chemical exchange saturation transfer to obtain richer dual-modality information. Lastly, our proposed method cannot be used in studies where simultaneous PAT/MRI acquisition is required, e.g. imaging of dynamic neural response, functional activation, perfusion of dual-modality probes, etc.

## 5 Conclusions

In this work, we have demonstrated the feasibility of an image acquisition and co-registration method for PAT and MRI. The design of the dual-modality animal imaging bed ensures that the deformation of the animal is within acceptable range when switching imaging modalities, thereby simplifying image co-registration. The dual-modality hybrid-contrast image obtained with our method simultaneously provides functional and structural information. This simple and reliable method can be widely implemented for various PAT-MRI dual-modality in vivo animal studies.

## CRediT authorship contribution statement

**Shuangyang Zhang**: Methodology, Software, Investigation, Data analysis, Writing – original draft. **Xipan Li:** Software. **Kaiyi Tang:** Software. **Zhichao Liang**: Software. **Xiaoming Zhang, Zongxin Mo, Jian Wu and Shixian Huang**: PAT Experiments. **Jiaming Liu and Zhijian Zhuang**: MRI Experiments. **Li Qi:** Conceptualization, Supervision, Funding acquisition, Writing – review & editing **Wufan Chen**: Conceptualization, Supervision, Funding acquisition, Writing – review & editing.

## Declaration of Competing Interest

The authors declare that they have no known competing financial interests or personal relationships that could have appeared to influence the work reported in this paper.

## Data Availability

Data will be made available on request.
